# Negative Pressure Incisional Therapy and Postoperative Infection after Posterior Approach Primary Total Hip Arthroplasty

**DOI:** 10.7759/cureus.7394

**Published:** 2020-03-24

**Authors:** Vineet Tyagi, Joseph Kahan, Patrick Huang, Don Li, David Gibson

**Affiliations:** 1 Orthopaedics, Yale New Haven Hospital, New Haven, USA; 2 Orthopaedics and Rehabilitation, Yale University School of Medicine, New Haven, USA

**Keywords:** orthopedics, hip, arthroplasty, infection, negative pressure

## Abstract

A major complication after total hip arthroplasty (THA) is infection, which can have devastating clinical and financial results. Silver-impregnated dry dressings, such as Aquacel dressings, and incisional negative pressure dressings (Prevena) have been developed to reduce the rates of surgical site infections (SSIs) after surgery. We retrospectively reviewed the medical records of 235 patients who underwent primary posterior approach THA at our institution during a three-year period. Patients were grouped based on surgical dressing. Rates of SSI were recorded, as well as the effects of factors including age, sex, body mass index, and medical comorbidities. In the high-risk subgroup, defined as BMI > 30 and ASA > 3, the infection rate was 2.97% in the Aquacel group, compared to 1.20% in the Prevena group. This difference did not reach statistical significance. There was a statistically significant impact on readmissions rate (p = 0.028) and reoperation (p = 0.001). The findings of this study suggest that negative pressure dressings in carefully selected patients may help to reduce reoperations and readmissions in this subgroup.

## Introduction

Total hip arthroplasty (THA) has been shown to be a successful procedure for degenerative changes of the hip, with over 95% survivorship with 10 years of follow-up [1, 2]. As the number of patients with hip osteoarthritis continues to increase, the need for THA is growing in the United States. It is estimated that nearly half a million primary THAs will be performed yearly by 2030 [2].

Infections after total hip arthroplasty can be difficult complications, both in terms of financial effects on the healthcare system and patient morbidity. The incident of prosthetic joint infection after primary THA is estimated to be around 1-2% [3-5]. Certain medical comorbidities, such as diabetes, rheumatoid arthritis, and obesity, serve as risk factors which predispose patients to infections [6-8]. With an aging population in the United States, these risk factors become more common, and as a result, complication rates after THA are expected to increase [9-13,14].

The financial implications of revision surgeries have been well documented. The cost of revision surgery has significantly increased in the past decade, from approximately $55,000 in 2006 to over $75,000 by 2013 [3,15]. The cumulative medical cost of revision arthroplasty surgery in the United States is estimated to exceed $1.62 billion by 2020 [16].

Closed incision negative pressure wound therapy (ciNPWT) has been used as a measure to decrease wound drainage, which could potentially help to reduce deep infection [17, 18]. These types of dressings have been historically used for complications, non-healing wounds, or as adjuncts between wound debridement and definitive surgical closure [19, 20]. Negative pressure dressings function to accelerate the healing process through a variety of mechanisms: increased vascular flow through angiogenesis, decreased edema, and induction of collagen transcription [21-23].

Currently, the use of negative pressure dressings is accepted for the prophylaxis of wound complications in closed surgical wounds in high risk patients. We have previously analyzed the efficacy of negative pressure dressings in primary anterior total hip arthroplasty [18]. There are no specific guidelines that outline indications for the use of incisional negative pressure dressings after primary posterior approach THA. Our goal is to determine preoperatively which patients have a higher risk of developing prolonged wound drainage and subsequent infection, so that we can help surgeons determine who may benefit from incisional negative pressure dressings. We hypothesized that there would be a lower postoperative surgical site infection (SSI) rate in high risk patients who were treated with a negative pressure incisional dressing (Prevena by Acelity / KCI, San Antonio, TX), compared to those with our current “standard” occlusive, antibacterial surgical site dressing (Aquacel by Convatec, Bridgewater, NJ).

## Materials and methods

Study design

The goal of this study was to compare infection rates between ciNPWT dressings (Prevena) and a silver-impregnated occlusive island dressing (Aquacel) after primary total hip arthroplasty done through a posterior approach. After receiving Institution Review Board (IRB) approval, we retrospectively reviewed the electronic medical records of 235 patients who underwent primary total hip arthroplasty through a posterior approach at our institution from January 2016 to January 2019.

Inclusion criteria included primary THA through a posterior approach at our institution by a single surgeon (DG). Exclusion criteria included revision hip arthroplasty, postoperative follow-up of less than three months, conversion of a hemiarthroplasty to THA, and age under 18 years. The primary outcome was development of any postoperative SSI. Secondary outcomes included length of stay (LOS), readmission, and need for reoperation. Age, sex, American Society of Anesthesiology (ASA) class, and type of surgical dressing use were determined by reviewing patient medical records. Additional data, including body mass index (BMI), tobacco use, history of autoimmune disease, preoperative International Normalized Ratio (INR), and procedure details, were also obtained directly from patient records.

Surgical technique

All THAs were performed by a single surgeon who used a posterior approach using the Pinnacle acetabular cup and either a press-fit Summit or S-ROM stem (Depuy Synthes, Warsaw, IN). The surgeon used Aquacel dressings on all of his patients until halfway through the study period (starting July 2018), when he began using Prevena ciNPWT on patients classified as high risk (BMI ≥ 30 and/or ASA class ≥ 3) and silver-impregnated island (Aquacel) dressing in the rest of the “standard risk” patients.

Statistical analysis

Patient and procedure characteristics were tabulated, and differences between the groups were assessed using Pearson’s chi-squared test, z score comparison between two proportions, unpaired student t-test between two means, or Fisher’s Exact Test to compare patient demographics, ASA class, and procedure types for patients based on dressing category. All calculations were performed using Stata 13.1 (StataCorp, College Station, TX) or Excel (Microsoft, Silicon Valley, CA), and the threshold for significance was a type I error rate of 0.05 adjusted if appropriate for multiple comparisons. A separate subgroup analysis was performed by risk stratifying patients into categorical “standard” and “high risk” patient groups, defined as patients with BMI ≥ 30 and/or ASA class ≥ 3, to determine whether high-risk patients would benefit from iNPWT dressings. Similar analyses were used for the high-risk group including multivariable linear regression modeling.

## Results

Between January 2016 and January 2019, 235 patients met the inclusion/exclusion criteria of our study and underwent a total of 235 total hip arthroplasties via the posterior approach. iNPWT was used in 92 patients and silver-impregnated occlusive dressing was used in 143 patients. Patient demographics and baseline characteristics are demonstrated in Table 1. There was no significant difference in age, history of diabetes, smoking status, or autoimmune disease between the two groups. Patients who received iNPWT had a higher BMI (39.5) than patients who received an occlusive dressing (29.6), p = <0.001. They also had a mean ASA class of 2.7, compared to 2.5 for the occlusive dressing group, p = <0.001.

**Table 1:** Demographics and baseline characteristic data for study groups BMI: Body mass index; ASA: American Society of Anesthesiologists.

Characteristic	Aquacel	Prevena	P-Value
Total Patients	143	92	
Average Age	64.8	61.9	0.098
% Male	42.6%	17.4%	<0.001
Average BMI	29.6	39.5	<0.001
Average ASA Class	2.5	2.7	0.008
% High Risk	70.6%	90.2%	<0.001
% Diabetes Diagnosis	23.1%	26.1%	0.601
% Smoker	51.0%	51.1%	0.988
% Autoimmune Diagnosis	13.3%	21.7%	0.092

As shown in Table 2, there was no difference in the rate of SSI between the two groups overall (p = 0.553). The overall SSI rate in this cohort of 235 cases was 1.28%. There was a trend toward lower overall readmission rate among the iNPWT group compared to the occlusive dressing group (6.52% vs. 10.49%), but it was not statistically significant (p = 0.43). There was a trend toward lower reoperation rates in the iNPWT group, but this was not statistically significant (p = 0.09). There was no difference in administration of tranexamic acid or length of stay between the two groups. Mean operative time of 89 minutes was longer in the Prevena group, p = 0.001.

**Table 2:** Surgical site infections, outcomes, and operative data between groups TXA: Tranexamic acid

Characteristic	Aquacel	Prevena	P-Value
Total Patients	143	92	
Surgical Site Infection	2% (n = 3)	1% (n = 1)	0.553
Readmission	10.49% (n = 15)	6.52% (n = 6)	0.430
Reoperation	10% (n = 14)	4% (n = 4)	0.092
% TXA Administered	85%	91%	0.178
Operative Time (minutes)	72	89	0.001
Length of Stay (days)	3.1	3.0	0.855

In our subgroup analysis of high-risk patients (Tables 3, 4), defined by BMI ≥ 30 and ASA ≥ 3, we found no difference in age, ASA class, history of diabetes, smoking status, or autoimmune diagnosis. Patients in the Prevena subgroup were more likely to be male (p < 0.001) and had a lower BMI of 40.6, compared to 31.2 in the Aquacel group (p < 0.001). In the high-risk subgroup, the infection rate was 2.97% in the Aquacel group, compared to 1.20% in the Prevena group. This difference did not reach statistical significance (p = 0.348). There was a lower reoperation rate in the Prevena group (13.86% vs 1.20%, p = 0.001), as well as readmissions (14.85% vs 4.82%, p = 0.028). The mean operative time was longer in the Prevena group (87.2 vs 75 minutes, p = 0.005). There was no difference in length of stay between the groups.

**Table 3:** Demographics and baseline characteristic data for high-risk subgroup BMI: Body mass index; ASA: American Society of Anesthesiologists.

Characteristic	Aquacel	Prevena	P-Value
Total Patients	101	83	
Average Age	65.5	62.1	0.064
% Male	45%	19%	<0.001
Average BMI	31.2	40.6	<0.001
Average ASA Class	2.8	2.8	0.817
% Diabetes Diagnosis	27%	29%	0.764
% Smoker	53%	54%	0.893
% Autoimmune Diagnosis	16%	19%	0.594

**Table 4:** Surgical site infections, outcomes, and operative data for high-risk subgroup TXA: Tranexamic acid

Characteristic	Aquacel	Prevena	P-Value
Total Patients	101	83	
Surgical Site Infection	2.97% (n = 3)	1.20% (n = 1)	0.348
Readmission	14.85% (n = 15)	4.82% (n = 4)	0.028
Reoperation	13.86% (n = 14)	1.20% (n = 1)	0.001
% TXA Administered	82%	90%	0.125
Operative Time (minutes)	75	87.2	0.005
Length of Stay (days)	3.22	3.02	0.620

Table 5 shows the results of multivariate regression. BMI and ASA class were the two variables (p = 0.043 and p = 0.034, respectively) associated with surgical site infection in our study.

**Table 5:** Multivariate regression with SSI as dependent variable BMI: Body mass index; ASA: American Society of Anesthesiologists; SSI: Surgical site infection.

Predictors	P-Value
Prevena	0.024
BMI	0.043
Age	0.746
Gender	0.754
ASA Class	0.034
Diabetes Mellitus	0.542
TXA	0.451
Smoking	0.531
Autoimmune	0.099

## Discussion

The objective of Prevena iNPWT is to reduce drainage and help decrease the risk of surgical site infections. All infections observed in this study occurred in the high-risk cohort. Our study suggests that the use of Prevena dressings in patients at higher risk of developing infections may help to reduce reoperation and readmissions, and that these patients may benefit from prophylactic use of Prevena dressings.

In patients at risk of developing infection, the use of NPWT has been shown to decrease the incidence of postoperative seroma and hematoma, as well as infection [24]. Research looking specifically at NPWT in orthopaedics is limited, especially in the subset of patients undergoing primary joint arthroplasty. Due to the potential costs associated with negative pressure dressings, understanding their possible benefits in preventing infections after THA is a worthwhile consideration. In the literature, the cited cost of a standard Prevena dressing is $495, compared to a standard Aquacel dressing at $15 [25, 26]. At our institution in June 2018, the cost of a Prevena dressing was $486, compared to $39 for an Aquacel dressing. Therefore, selective use of Prevena dressings is important.

Pachowsky et al. conducted a randomized controlled trial and found that prophylactic negative pressure dressings after primary THA reduced the volume of postoperative seromas [27]. The study did not analyze prosthetic joint infection [27]. Karlakki et al. found that NPWT in primary total hip and knee arthroplasty led to decreased wound exudate and related complications, as well as lower LOS [28]. Patients in the NPWT group also had fewer dressing changes. The study also found that there were fewer dressing changes, as expected, in the NPWT group. No financial analysis was completed, but subgroup analysis found reductions in wound complications for higher risk patients with an ASA ≥ 3 and a BMI ≥ 35 [28]. Siqueira et al. confirmed a literature review and found similar results that NPWT may be preferentially beneficial in high-risk patients. The review identified high risk of bleeding/hemorrhage postoperatively, patient size/weight, and intraoperatively tissue quality as patient factors that may predispose patients to infection after primary THA [29].

Redfern et al. performed a study comparing a historical control group treated with gauze dressings after THA and total knee arthroplasty (TKA) to a prospective cohort of patients treated with NPWT. There was no difference in deep infections between the groups, but there was a lower rate of superficial infections in the NPWT group. The important caveat for this study was a significant difference in baseline characteristics such as gender, surgery site, and medical comorbidities between the two groups. The study also used plain gauze dressings instead of silver-impregnated antimicrobial dressings [30].

One of the primary limitations of this study is its retrospective design, which limits the ability to make broad conclusions. A future prospective randomized controlled study would be needed to further study the effects of dressings on postoperative infections, especially for high-risk patients. The study of a single surgeon’s (DG) cases, designed to eliminate variability in patient selection and surgical technique, limits broader applicability. Analysis of a larger patient cohort may have allowed increased data collection and strengthened results. Future randomized studies with more surgeons are needed to better understand the role of NPWT in primary posterior approach THA.

To our knowledge, this is the first study examining NPWT in a large cohort of primary posterior approach THA. There are no disclosures or conflicts of interest related to either of the dressings used for any of the study authors. Despite the noted limitations of the study, we feel the results reported constitute an important addition to the literature.

## Conclusions

Our findings suggest that the use of incisional NPWT helps to reduce reoperation and readmission rates in high-risk patients after undergoing primary posterior approach THA. There difference in infections did not reach statistical significance, likely due to limitations in study size. Given the significant costs associated with reoperations and readmissions, our data indicates that prophylactic use of iNPWT in high-risk patients may be a useful adjunct to reduce complications. As a result of this data, we have continued to stratify patients and use incisional negative pressure dressings in those patients deemed to have a higher risk of developing infections. Further study is needed regarding the use of incisional NPWT dressings in high risk and revision hip arthroplasty cases.
